# Aggression and Risk Behaviors in a Group of Adolescents with High-Functioning Autism

**DOI:** 10.3390/children12070852

**Published:** 2025-06-27

**Authors:** Mihaela Moise, Lucia Emanuela Andrei, Ilinca Mihailescu, Alexandra Mariana Buica, Florina Rad

**Affiliations:** 1 Child and Adolescent Psychiatry Department, “Carol Davila” University of Medicine and Pharmacy, 020021 Bucharest, Romania; mihaela.stancu@drd.umfcd.ro (M.M.);; 2 Psychiatry Department, “Mina Minovici” National Institute of Legal Medicine, 042122 Bucharest, Romania; 3 Child and Adolescent Psychiatry Department, “Prof. Dr. Alexandru Obregia” Clinical Psychiatry Hospital, 041914 Bucharest, Romania

**Keywords:** autism spectrum disorder, high-functioning autism, adolescents, aggression, anger regulation, conduct disorder, emotion dysregulation, STAXI-2, trait anger

## Abstract

Background/Objectives: Aggression in adolescents with autism, particularly those with high-functioning autism (HFA), presents a unique clinical profile. The aim of this study was to assess and compare anger expression and regulation in adolescents with HFA, those diagnosed with conduct disorder (CD), and a control group with no psychiatric diagnoses. Methods: A total of 120 adolescents aged 14–17 were divided into three equal groups: 40 with HFA, 40 with CD, and 40 controls. Participants were assessed using the State-Trait Anger Expression Inventory (STAXI-2), which measures emotional intensity, trait predisposition, and modes of anger expression and control. Non-parametric statistical analyses were conducted to examine group differences. Results: Statistically significant differences were found across most STAXI-2 scales. Adolescents with CD exhibited the highest scores on anger intensity and expression, followed—at a lower level—by those with HFA. The autism group showed significantly elevated levels of verbal anger expression and frustration reactivity compared to controls, but lower tendencies for physical aggression. Trait anger was also higher in the HFA group, particularly in response to frustration or criticism. No significant differences were found between the HFA and control groups on anger control scales individually; however, the overall Anger Expression Index was significantly elevated in HFA, reflecting a global imbalance between anger expression and regulation. Conclusions: Adolescents with high-functioning autism exhibit a distinct profile of emotional dysregulation, characterized by increased verbal anger and frustration sensitivity, despite lower levels of overt aggression. This comparative pilot study contributes to a better understanding of emotional dysregulation and anger expression in adolescents with HFA. These findings highlight the need for tailored emotion regulation interventions. School-based programs focused on emotional awareness and verbal anger management could offer meaningful benefits for this population. Future research should expand sample diversity, explore gender differences, include common comorbidities like ADHD, and investigate longitudinal and neurobiological patterns of anger regulation in ASD.

## 1. Introduction

Aggression in adolescence is a multidimensional phenomenon influenced by neurobiological, psychological, and environmental factors, and it can manifest through a variety of expressions, from irritability and verbal hostility to physical violence [1,2].

Clinical practice and empirical studies increasingly document aggressive behavior in individuals with ASD, including those with high-functioning autism (HFA). According to the Diagnostic and Statistical Manual of Mental Disorders, Fifth Edition, Text Revision (DSM-5-TR; APA, 2022), autism spectrum disorder (ASD) is characterized by persistent deficits in social communication and social interaction across multiple contexts, along with restricted, repetitive patterns of behavior, interests, or activities [3]. These symptoms must be present from early developmental stages, cause clinically significant impairment, and not be better explained by intellectual disability or global developmental delay. HFA is characterized by intact intellectual capacities and, frequently, well-developed language skills, which distinguish this subgroup from individuals with more severe autism [3].

Understanding aggressive behaviors in adolescents with autism spectrum disorders (ASD), particularly those with HFA, requires complex research into the relationship between neurodevelopmental factors, emotional regulation difficulties, and environmental stressors. Although aggression is not a core diagnostic criterion for ASD, it is frequently reported in clinical settings and can have a significant impact on the individual’s social integration, academic performance, and family dynamics [4,5].

Emotional dysregulation, comorbid psychiatric conditions, and deficits in executive functioning are interrelated factors that significantly contribute to aggressive behavior in adolescents with HFA. Emotion dysregulation often manifests as heightened irritability and reactive aggression in response to environmental or social stressors [6,7]. Comorbidities such as anxiety, ADHD, and depression further exacerbate emotional instability, complicating the clinical presentation [8,9]. Executive dysfunction—including poor impulse control, cognitive inflexibility, and limited attentional shifting—reduces adaptive coping and increases the risk for maladaptive responses such as verbal or physical aggression. Together, these factors represent core targets for both assessment and intervention [10,11,12].

Neurobiological studies further underline these patterns by suggesting atypical features in amygdala and prefrontal cortex connectivity in individuals with HFA, which could potentially contribute to increased emotional reactivity and impaired regulatory control. These neurological differences provide a basis for understanding the behavioral phenotypes observed in this population [13,14].

The comparison between adolescents with HFA and with CD is clinically meaningful. While both groups may exhibit externalizing behaviors, their etiologies and presentation can differ significantly. CD is associated with early-onset disruptive behavior, lack of empathy, and persistent violation of social norms [15]. In contrast, adolescents with HFA typically do not exhibit callous-unemotional traits; rather, their aggressive behavior is context-dependent, reactive, and often accompanied by emotional distress, anxiety, or confusion in response to perceived threats or misunderstandings [16].

Finally, it is essential to take into consideration the role of environmental and family influences. Parenting styles, peer victimization, and school accommodations (or lack thereof) can have a significant impact on the occurrence and frequency of aggressive episodes. Adolescents with HFA often experience social rejection or aggression, which can intensify their frustration and feelings of alienation, leading to externalizing responses [17,18].

Despite increased awareness, few studies have systematically compared emotional and behavioral profiles across HFA, CD, and non-clinical groups using standardized tools. This study aims to address that gap. The present study is a non-interventional, comparative pilot investigation aimed at identifying patterns of emotion regulation and aggression in adolescents with high-functioning autism relative to two matched groups. Given the modest sample size, the current project should be considered a pilot study aimed at identifying preliminary patterns in emotional dysregulation among adolescents with HFA.

## 2. Materials and Methods

### 2.1. Participants

This study included three groups of adolescents with ages between 14–18 years old: 40 adolescents diagnosed with an autistic spectrum disorder (specifically high-functioning autism), 40 adolescents diagnosed with conduct disorder (CD), and a control group of 40 adolescents without psychiatric diagnoses.

Participants were recruited through convenience sampling from the Child and Adolescent Psychiatry Department of the “Prof. Dr. Alexandru Obregia” Clinical Psychiatry Hospital. The study included two groups of adolescents with distinct psychiatric diagnoses: Autism Spectrum Disorder (particularly high-functioning autism, HFA) and conduct disorder (CD). Group allocation was based on clinical diagnoses established by specialized psychiatrists according to DSM-5-TR criteria. The HFA group consisted of 40 adolescents diagnosed with autism spectrum disorder, without intellectual disability (IQ > 70), without language delay, and with no comorbid diagnosis of conduct disorder. The CD group included 40 adolescents diagnosed with conduct disorder, also without intellectual disability (IQ > 70), without language delay, and without a comorbid diagnosis of autism spectrum disorder. Exclusion criteria for both groups included: comorbidity of HFA and CD; diagnosis of attention-deficit/hyperactivity disorder (ADHD), due to its strong association with increased impulsivity, emotional dysregulation, and reactive aggression, which could confound the results of this study; intellectual disability (IQ < 70); language delay; any additional neurological or developmental disorder that could impair comprehension of study instructions; refusal to participate by the adolescent or their parent/legal guardian. Participants in the HFA and CD groups were recruited consecutively during clinical assessments conducted at the Child and Adolescent Psychiatry Department of the “Prof. Dr. Alexandru Obregia” Clinical Hospital between 2019 and 2023. The control group consisted of 40 adolescents recruited from middle and high schools (grades 8 through 12, according to the Romanian educational system). Control group participants were recruited from mainstream schools in the same urban area and were matched by age and gender to the clinical groups. Matching for socioeconomic status or school type was not formally performed. Inclusion criteria required the absence of any psychiatric diagnosis or prior contact with psychiatric services. Participants also exhibited no clinically evident intellectual disability, language delay, or any self-reported neurological or developmental disorder that could interfere with their ability to comprehend study instructions. Clinical data for adolescents with HFA and CD were collected during evaluations at the Child and Adolescent Psychiatry Department of the “Prof. Dr. Alexandru Obregia” Clinical Psychiatry Hospital. These assessments were performed in designated interview rooms, and parents or legal guardians were present during consent procedures and were involved in providing complementary information. For the control group, data collection took place in designated rooms within the schools, under supervision of the researchers and with parental consent. Adolescents completed the self-report questionnaires on-site.

A demographic analysis was performed to examine the gender distribution across the three groups. The autism group consisted of 14 females (35%) and 26 males (65%). The conduct disorder group included 16 females (40%) and 24 males (60%), while the control group was comprised of 17 females (42.5%) and 23 males (57.5%).

The ages of the adolescents in all three study groups ranged from 14 to 17 years. The mean age for the group diagnosed with conduct disorder was 15.38 years (SD = 1.10). The group of adolescents with autism spectrum disorder had a slightly higher mean age of 15.58 years (SD = 0.84), identical to that in the control group: 15.58 years (SD = 0.98).

Participant enrollment was conducted between 2019 and 2023. Confidentiality and data anonymization were maintained through the use of secure digital records. The study received ethical approval from the hospital’s ethics committee, and all data were handled in accordance with institutional confidentiality policies and ethical research standards.

### 2.2. Instruments

**Aggression Risk Assessment—Questionnaires used**

STAXI-2 (State-Trait Anger Expression Inventory, 2nd Edition).

The STAXI-2 is a widely used psychological tool designed to measure the intensity of anger as an emotional state (state anger), the disposition to experience angry feelings over time (trait anger), and the manner in which anger is expressed or controlled (Anger Expression and Anger Control scales). This instrument helps differentiate between temporary and persistent anger tendencies and evaluates how individuals express and manage anger. STAXI is comprised of multiple scales: S_ang (State Anger); S_ang_f (Feeling Angry); S_ang_v (Feel Like Expressing Anger Verbally); S_ang_p (Feel Like Expressing Anger Physically); T_ang (trait anger); T_ang_t (Angry Temperament); T_ang_r (Angry Reaction); Ax_o (Anger Expression-Out); Ax_i (Anger Expression-In); Ac_o (Anger Control-Out); Ac_i (Anger Control-In); Ax_index (Anger Expression Index). The instrument demonstrated high internal consistency in our sample (Cronbach’s alpha = 0.876).

### 2.3. Procedure

Data collection. For participants in the clinical groups, data were obtained from clinical records and supplemented by information provided by parents or legal guardians. For the control group, data were collected through self-report questionnaires completed by both the adolescents and their parents. Collected data included demographic variables such as age, gender, educational level, and history of criminal behavior or encounters with law enforcement. Medical data included psychiatric diagnosis, comorbidities, presence of substance use, IQ level and other relevant medical information. Information regarding prior criminal behavior or interactions with law enforcement was obtained via self-report and corroborated, where possible, with clinical records.

Researchers ensured that all participants fully understood the questionnaire items. For younger participants or those who requested clarification, items were read aloud and explained using standardized, non-leading prompts to maintain validity.

### 2.4. Data Analysis

For the purposes of this study, analysis focused on the results of the STAXI-2 (State-Trait Anger Expression Inventory—2nd Edition) questionnaire. The primary objective of the statistical analysis was to compare scores across the three study groups: adolescents diagnosed with a high-functioning autism spectrum disorder (HFA), those with conduct disorder (CD), and a control group without psychiatric diagnoses.

In order to select the most appropriate statistical approach for group comparisons, the distribution of scores on each STAXI scale was initially examined across the three groups. Normality was assessed using the Shapiro–Wilk test, performed separately for each scale within each group. The results revealed significant deviations from normality in several variables, with no scale demonstrating a normal distribution across all groups. In addition, Levene’s test for homogeneity of variances indicated violations in key comparisons (e.g., S_ang). Given the relatively small sample size per group (*n* = 40) and the non-normal distribution of several variables, we used non-parametric statistical tests to examine group differences. The Kruskal–Wallis H test was used to assess group differences on all STAXI scales. For scales where significant group effects emerged, post hoc analyses were performed using Mann–Whitney U tests, with Bonferroni corrections applied as appropriate to control for multiple comparisons. Effect sizes (r) were calculated using the formula r = Z/√N, where Z-scores were derived from the Mann–Whitney U tests and N = 80 (40 per group).

## 3. Results

**Figure 1:**
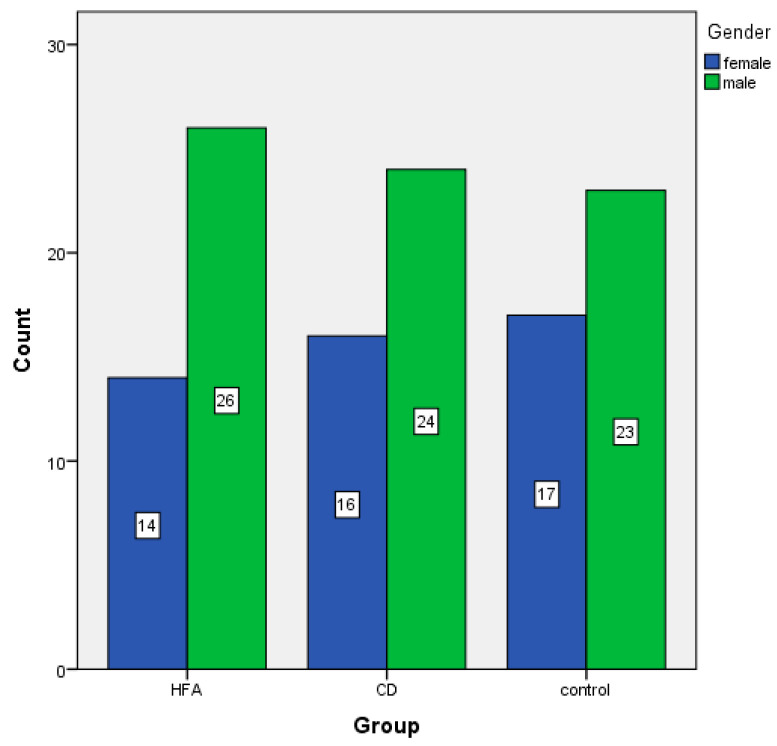
Gender distribution among groups.

A Chi-square test revealed no statistically significant differences in gender distribution between groups, χ^2^(2) = 0.490, *p* = 0.783. These results suggest that gender was distributed similarly across all three groups and that any observed variation is likely attributable to random variation rather than systematic difference. The gender distribution between groups is shown in Figure 1.

**Figure 2:**
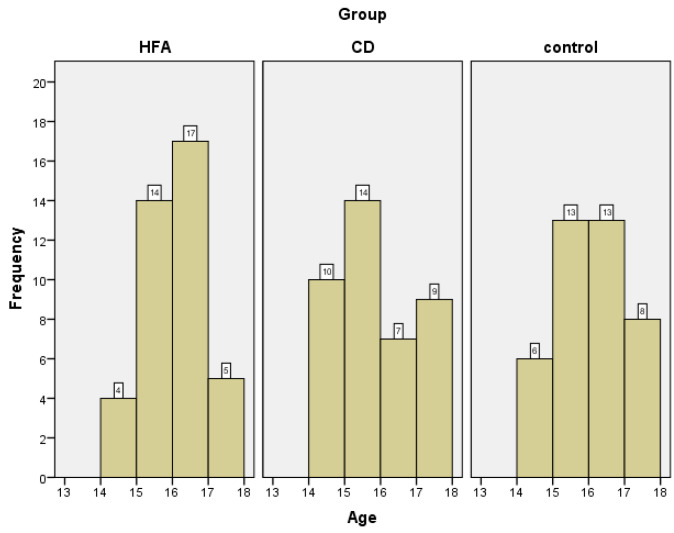
Age distribution between groups.

To examine potential differences in mean age between groups, a Kruskal–Wallis H test was performed. Although the mean ages were similar across groups (ranging from 15.38 to 15.58 years), the results indicated no statistically significant difference, H(2) = 1.29, *p* = 0.525. The age distribution in the three groups is illustrated in Figure 2.

The Kruskal–Wallis H test was performed to assess differences between the three groups—adolescents with conduct disorder, a control group, and individuals with autism spectrum disorders—on each of the STAXI scales. The analysis revealed statistically significant differences on all scales.

S_ang and its subscales—Feeling Angry (S_ang_f), Feel Like Expressing Angry Verbally (S_ang_v), and Feel Like Expressing Angry Physically (S_ang_p)—showed significant differences between groups (Table 1). Mann–Whitney U tests with Bonferroni correction revealed the highest scores in the conduct disorder (CD) group, followed by the high-functioning autism (HFA) group, and the lowest in the control group. For the overall S_ang scale, the HFA group scored significantly higher than the control group (U = 349.00, *p* = <0.001, r = 0.486) and significantly lower than the CD group (U = 525.00, *p* = 0.008, r = 0.297); the CD group, scored markedly higher than controls (U = 14.00, *p* < 0.001, r = 0.847). On the S_ang_f subscale (Feeling Angry), HFA participants again scored significantly higher than controls (U = 315.50, *p* < 0.001, r = 0.527) and differed from the CD group as well (U = 535.00, *p* = 0.010, r = 0.288); the CD group showed substantially higher scores than controls (U = 32.00, *p* < 0.001, r = 0.832). On the S_ang_v subscale (verbal anger expression), HFA individuals scored significantly higher than controls (U = 397.50, *p* = <0.001, r = 0.436), and while the difference with the CD group did not reach statistical significance (U = 604.00, *p* = 0.058), CD participants scored significantly higher than controls (U = 58.50, *p* < 0.001, r = 0.802). Finally, for S_ang_p (physical anger expression), the HFA group did differ significantly from controls (U = 553.50, *p* = 0.017, r = 0.268) as well as from the CD group (U = 443.50, *p* = 0.001, r = 0.387); the CD group again displayed the highest scores, significantly above the control group (U = 72.50, *p* < 0.001, r = 0.789).

The T-Ang scale measures how frequently feelings of anger are expressed over time. It includes two subscales: Angry Temperament (T_ang_t), which assesses the tendency to experience anger without a specific trigger, and Angry Reaction (T_ang_r), which measures how often anger arises in response to frustration or negative feedback. Trait anger (T_ang), along with its subscales, showed significant group differences (Table 2). Scores were highest in the conduct disorder (CD) group, followed by the autism (HFA) group, and lowest in the control group. For the overall T_ang scale, HFA adolescents scored significantly higher than controls (U = 313.00, *p* < 0.001, r = 0.526), and CD adolescents scored significantly higher than the control group (U = 36.50, *p* < 0.001, r = 0.825). There was no statistical difference between the scores of HFA and CD groups (U = 622.50, *p* = 0.086). On the Angry Reaction subscale (T_ang_r), the HFA group again scored significantly higher than controls (U = 329.00, *p* < 0.001, r = 0.513) and differed from the CD group (U = 501.00, *p* = 0.004, r = 0.326), while the CD group scored highest overall (CD vs. control: U = 78.50, *p* < 0.001, r = 0.785). In contrast, for the Angry Temperament subscale (T_ang_t), the CD group showed significantly elevated scores compared to controls (U = 148.00, *p* < 0.001, r = 0.712); the difference between HFA and control was also significant (U = 407.50, *p* < 0.001, r = 0.426), but HFA and CD did not differ significantly (U = 742.50, *p* = 0.575).

The Anger Expression-Out (Ax_o), Anger Expression-In (Ax_i), Anger Control-Out (Ac_o), Anger Control-In (Ac_i), and Anger Expression Index (Ax_index) scales revealed mixed patterns of group differences (Table 3). For Ax_o, the conduct disorder (CD) group scored significantly higher than the control group (U = 297.00, *p* < 0.001, r = 0.546), while the autism (HFA) group also differed significantly from controls (U = 491.50, *p* = 0.003, r = 0.334); no significant difference was found between the HFA and CD groups (U = 747.50, *p* = 0.610). The Anger Expression-In scale (Ax_i) assesses the tendency to suppress feelings of anger rather than expressing them outwardly. On this scale, the CD group scored significantly higher than the control group (U = 406.50, *p* < 0.001, r = 0.431). However, there were no significant differences between the CD and HFA groups (U = 701.00, *p* = 0.338, r = 0.107), nor between the HFA and control groups (U = 651.00, *p* = 0.148, r = 0.162). In terms of Ac_o, the scores for CD group differed significantly from the scores in the control group (U = 537.00, *p* = 0.010, r = 0.286), and from those in the HFA group (U = 568.00, *p* = 0.025, r = 0.251), but differences between HFA and control (U = 690.00, *p* = 0.286) were not significant. Regarding Ac_i, the CD group again differed significantly from controls (U = 404.50, *p* < 0.001, r = 0.431), and from HFA subjects (U = 557.50, *p* = 0.019, r = 0.263), while comparisons involving the HFA group and the control group (U = 796.50, *p* = 0.973) were not statistically significant. Finally, the Ax_index revealed significantly higher scores in the HFA group compared to controls (U = 458.50, *p* = 0.001, r = 0.369). HFA also differed significantly from the CD group (U = 578.00, *p* = 0.032, r = 0.240), while no significant difference was observed between CD and control (U = 773.00, *p* = 0.794).

To explore potential gender differences in anger expression and regulation, Mann–Whitney U tests were conducted across all STAXI-2 scales. Gender-based differences in anger expression and regulation were explored separately within each group (Table 4). In the HFA group, a significant difference was observed on the Anger Control-Out (Ac_o) scale (*p* = 0.047), with boys reporting higher levels of control over outward expressions of anger than girls. In the CD group, only one significant gender difference was found: boys scored higher on Anger Control-In (Ac_i) than girls (*p* = 0.023), suggesting greater self-reported internal regulation of anger. In the control group, several significant differences emerged. Boys reported higher scores on Feeling Angry (S_ang_f) and Anger Expression Index (Ax_index), while girls scored higher on trait anger (T_ang), Angry Reaction (T_ang_r), and Anger Control-In (Ac_i). Additionally, non-significant trends approaching statistical significance were found for state anger (S_ang, *p* = 0.072) and physical anger expression (S_ang_p, *p* = 0.071) in the control group, with higher scores observed in boys. No other scales showed significant gender differences within any of the three groups.

## 4. Discussion

This discussion section interprets the findings of the current study, which aimed to compare anger expression and regulation in adolescents with high-functioning autism (HFA), conduct disorder (CD), and typically developing peers.

We first examined the state anger (S_ang) scale and its subcomponents: Feeling Angry (S_ang_f), Feel Like Expressing Anger Verbally (S_ang_v), and Feel Like Expressing Anger Physically (S_ang_p). The analysis revealed statistically significant differences between the three groups. Pairwise comparisons using the Mann–Whitney U test indicated that overall state anger was highest in the CD group, followed by the HFA group, and lowest in controls. Effect sizes ranged from r = 0.297 (HFA vs. CD) to r = 0.847 (CD vs. control), indicating moderate to very large differences.

On the Feeling Angry (S_ang_f) subscale, adolescents with conduct disorder reported the highest levels of anger, followed by those with high-functioning autism (HFA), and then the control group. Pairwise comparisons revealed that both clinical groups scored significantly higher than the control group. Moreover, the HFA group also differed from the CD group, showing lower scores on outward anger expression, although the difference was more modest. These results highlight an elevated tendency to experience anger in both clinical populations, but more pronounced in conduct disorder. The pattern reflects the broader emotional dysregulation associated with externalizing disorders, while also pointing to notable affective difficulties among autistic adolescents.

The Feel Like Expressing Anger Verbally (S_ang_v) subscale showed significant differences between the conduct disorder and control groups, and between the control and autism groups. However, no statistically significant difference was found between the conduct disorder and autism groups (*p* = 0.058), although descriptive scores suggest that adolescents with conduct disorder may still report slightly higher verbal anger expression. This may indicate that autistic adolescents also experience elevated verbal anger, potentially reflecting difficulties with frustration tolerance, communication, or emotional modulation.

Regarding the Feel Like Expressing Anger Physically (S_ang_p) subscale, adolescents in the conduct disorder group reported the highest levels of physical anger expression, clearly exceeding those of both the HFA and control groups. The HFA group also reported slightly higher levels than the control group. These findings highlight a marked tendency toward physical expressions of anger among individuals with conduct disorder, but also suggest a mildly elevated risk for physical anger expression among adolescents with autism.

Taken together, these findings for the state anger scale suggest that adolescents with autism spectrum disorder exhibit a notably heightened anger profile compared to typically developing controls, particularly in the emotional and verbal expression dimensions of anger. Although their scores on the state anger (S_ang) scale and its subcomponents did not surpass those of adolescents with conduct disorder, they were consistently and significantly higher than those of the control group. However, in some cases—such as verbal anger expression (S_ang_v)—although the HFA group scored lower than the conduct disorder group, the difference did not reach statistical significance. This pattern underscores the affective vulnerability present in autism, which may not always manifest through physical aggression but can involve intense subjective anger and maladaptive expression strategies.

These findings are consistent with prior research indicating that emotion dysregulation is a core feature in ASD, particularly in adolescents and high-functioning individuals. Mazefsky et al. highlighted in 2014 that youth with autism frequently experience intense emotional responses, including anger, that are poorly modulated and can interfere with adaptive functioning [6]. Similarly, Laurent and Rubin (2004) noted that children and adolescents with ASD may display emotion regulation impairments comparable in severity to those observed in externalizing disorders, even if behavioral manifestations differ [19].

Our findings support the idea that adolescents with HFA may display distinct subtypes of aggressive behavior, particularly verbal or reactive forms, which contrast with the more impulsive and physical aggression observed in CD. Kirst et al. (2022) proposed that in ASD, specific subtypes of aggression are closely linked to deficits in emotion recognition, hostile attribution bias, and dysfunctional emotion regulation patterns—mechanisms that may underlie the verbal and reactive aggression found in our sample [20]. Incorporating such frameworks may enhance the specificity of future diagnostic assessments and interventions. Importantly, although conduct disorder is usually associated with externalized anger and impulsive aggression [21], the overlap in anger experience between autism and youth with conduct disorder in our study—particularly in the verbal domain—suggests that anger regulation difficulties in ASD may be underestimated if attention is focused exclusively on overt behavioral aggression.

This comparison emphasizes the need for targeted emotion regulation interventions not only for conduct disorder but also for adolescents with high-functioning autism, particularly those who present with verbal aggression, internalized anger, or low tolerance to frustration. As highlighted by Pugliese et al. (2015), addressing anger rumination and emotional awareness in HFA may be essential for reducing the psychosocial burden associated with dysregulated anger in this population [22].

The trait anger (T_ang) scale, which examines how frequently feelings of anger are experienced over time, showed a clear differentiation among groups. Adolescents with conduct disorder scored the highest, followed by those with autism, and then the control group. Pairwise comparisons revealed significant differences between the conduct disorder and control groups, and between the autism and control groups. However, the difference between the conduct disorder and autism groups was not statistically significant, indicating that adolescents with autism may exhibit a general propensity toward anger that approaches, but does not match, the levels observed in conduct disorder. These differences reflect large to very large effects in trait anger between groups and may indicate chronic emotional dysregulation in autism, potentially related to difficulties with frustration tolerance or social information processing.

The Angry Temperament (T_ang_t) subscale, which assesses the tendency to feel anger without an external trigger, showed that adolescents with conduct disorder reported the highest levels of impulsive, unprovoked anger, clearly exceeding those of both the autism and control groups. Adolescents with autism also reported a moderately elevated tendency compared to controls, suggesting that while such anger is most pronounced in conduct disorder, it is also relevant—though to a lesser extent—in autism. This pattern may reflect a combination of situational vulnerability and cognitively mediated responses in the HFA group.

In contrast, the Angry Reaction (T_ang_r) subscale, which measures how often anger arises in response to frustration or criticism, revealed a distinct pattern across groups. Adolescents with conduct disorder scored highest, followed by those with autism, while the control group scored the lowest. The autism group reported more reactive anger than controls, but somewhat less than the CD group, suggesting that heightened sensitivity to perceived frustration or criticism is present in both clinical groups, though more intense in conduct disorder. Such a pattern could be tied to difficulties in emotion regulation and interpreting social cues. Research by Samson et al. (2015) supports this, reporting that autistic adolescents show heightened anger responses when faced with social rejection or negative evaluation, which may result from difficulty understanding social nuances or reduced coping flexibility [23].

Taken together, these findings suggest that while adolescents with conduct disorder consistently exhibit the highest levels of trait anger and impulsive expression, adolescents with high-functioning autism also display significantly elevated anger, particularly in response to interpersonal stress and frustration. Although their trait anger scores do not reach the levels observed in conduct disorder, the HFA group still shows a heightened tendency toward anger relative to controls. This elevated trait anger profile in autism reflects underlying emotion regulation difficulties, potentially linked to low frustration tolerance and impairments in processing social feedback. These findings are in line with existing research, which emphasizes that emotion dysregulation is not only common in ASD but often severe, manifesting as intense, persistent emotional responses—including anger—that can be internally distressing and externally disruptive. These findings highlight the need to conceptualize emotion regulation as an important factor influencing functional outcomes in autism [24,25].

The analysis of anger regulation and expression through the Ax_o, Ax_i, Ac_o, Ac_i, and Ax_index scales revealed nuanced patterns in how anger is expressed or managed across the three groups.

On the Anger Expression-Out (Ax_o) scale, which measures the frequency with which anger is expressed outwardly toward others or objects, adolescents with conduct disorder scored significantly higher than those in the control group, indicating a pronounced tendency toward externalized anger. The HFA group also differed significantly from controls, though with a smaller effect size. However, no significant difference was found between the conduct disorder and autism groups, suggesting that autistic adolescents express outward anger at levels that fall between those of typically developing and conduct-disordered peers.

On the Anger Expression-In (Ax_i) scale, which assesses the extent to which individuals suppress or hold in their feelings of anger rather than expressing them outwardly, adolescents with conduct disorder scored significantly higher than those in the control group, indicating a greater tendency toward internalized anger expression in this population. In contrast, the autism group did not differ significantly from either the conduct disorder or the control group, suggesting that autistic adolescents may display more heterogeneous patterns of internal anger management. These findings imply that while internalized anger is notably elevated in conduct disorder, it may not represent a central feature of anger dysregulation in autism, where other dimensions such as verbal or reactive anger are more prominently affected.

On the Anger Control-Out (Ac_o) scale, which assesses an individual’s ability to inhibit the outward expression of anger, adolescents with conduct disorder surprisingly scored significantly higher than both the autism group and the control group, indicating a higher self-reported capacity for managing outward anger expression. No significant difference was observed between the autism and control groups. These results are somewhat counterintuitive given the well-established association between conduct disorder and externalizing, impulsive behaviors. One possible interpretation is that adolescents with CD may overestimate their ability to control their anger, either due to limited emotional insight or the tendency to underreport difficulties—intentionally or not—on self-report measures such as the STAXI-2. Another possibility is that youth with CD may exercise strategic, instrumental control over their anger, suppressing its expression in specific contexts while still engaging in aggressive behavior when it serves a purpose. In contrast, adolescents with autism may struggle more with real-time emotional awareness and modulation, leading to lower perceived levels of control, despite lower rates of overt aggression. This distinction underscores the complexity of anger regulation across diagnostic categories and the need to interpret self-report data in conjunction with observed behavior and clinical context.

On the Anger Control-In (Ac_i) scale, which reflects the ability to regulate internal feelings of anger, adolescents with conduct disorder scored significantly higher than both the control group and the autism group, indicating stronger self-reported capacity for managing internal anger. No significant difference was found between the autism and control groups, suggesting similar levels of internal anger control in these two groups. While these results may seem counterintuitive, they may reflect either overestimation of self-control in the CD group or a tendency to underreport emotional difficulties. Another possibility is that adolescents with CD develop compensatory self-monitoring strategies or emotional numbing that enhance perceived internal regulation. In contrast, autistic youth may report similar levels of internal control as non-clinical peers, despite potential difficulties with emotional insight or expression, highlighting the need for multi-informant assessment in this domain.

The Anger Expression Index (Ax_index), a composite measure reflecting the overall balance between anger expression and control, showed that adolescents with autism scored significantly higher than those in the control group, indicating moderately elevated difficulties in managing anger overall. Although not all individual subscales reached statistical significance—for instance, the HFA group did not significantly differ from controls on the Anger Control scales—the elevated total index suggests a cumulative dysregulation pattern, with subtle deficits in both expression and regulation contributing to an overall imbalance in anger management. This finding underscores the importance of considering global emotional regulation profiles rather than relying solely on isolated subscales when evaluating anger-related difficulties in autism.

The pattern of gender-based differences in anger regulation varied across groups. In the HFA group, boys reported higher levels of outward anger control than girls, which may reflect gendered socialization patterns, greater suppression of emotional expression in boys, or potential overestimation of self-control in male respondents. In the CD group, boys reported significantly better internal anger control, possibly suggesting that girls with conduct disorder experience more internal distress or have greater difficulty managing internalized anger. In the control group, several gender differences aligned with previous research: boys showed higher levels of state anger expression, while girls reported more internal anger regulation and higher trait anger reactivity. These findings suggest that gender-specific emotional processing styles may manifest differently depending on diagnostic context, and that both clinical and non-clinical interventions should be sensitive to such differences. While our study found only modest gender-based differences in anger expression and control within the HFA group, the existing literature suggests that such differences may be more pronounced in larger or more diverse samples. Neuhaus et al. (2022) emphasized the interactive effects of gender and language proficiency on aggression profiles in youth with ASD, noting that boys and girls may exhibit distinct pathways toward emotional dysregulation and outward behavior [26]. These perspectives reinforce the need for broader, more detailed studies to explore the nuanced influence of gender patterns on emotional regulation in autism. In parallel, therapeutic approaches should be tailored to reflect these gender-based emotional profiles, ensuring that interventions target the specific regulation challenges and expression styles typically observed in boys versus girls with ASD [26].

These findings align with existing literature indicating that individuals with high-functioning autism often experience significant challenges in emotion regulation, which can manifest through heightened reactivity, poor frustration tolerance, and difficulties in managing anger. Hendrix et al. (2022) emphasized that emotion regulation is a critical component in parent-mediated interventions for children with autism spectrum disorder (ASD), though it remains under-assessed and inconsistently targeted in early interventions [24]. Similarly, Davico et al. (2022) found that emotional dysregulation in preschoolers with ASD is strongly associated with poorer adaptive functioning, highlighting the urgent need for early and structured emotional support [25]. Factor, Swain, and Antezana (2019) demonstrated the efficacy of a structured cognitive-behavioral program—the Stress and Anger Management Program (STAMP)—in improving emotional awareness and reducing anger outbursts in young children with ASD [27]. Collectively, these studies underscore the importance of addressing emotion regulation systematically and early, using structured, developmentally appropriate interventions that can be adapted to the individual needs of autistic youth. Programs grounded in CBT principles, particularly those that incorporate parental involvement and emotional literacy components, appear especially promising for reducing anger and enhancing emotional self-regulation in this population [24,25,27].

Taken together, these results suggest that adolescents with conduct disorder demonstrate the most overt and impulsive difficulties in anger expression and regulation, while those with autism show a more complex emotional profile—characterized by increased verbal expression and frustration sensitivity, but without consistent deficits across all regulation domains. Compared to typically developing peers, autistic adolescents display a measurable imbalance between expression and control, as reflected by the elevated Anger Expression Index. Given the distinct emotional and behavioral profiles of the two clinical groups, intervention strategies should be tailored accordingly: adolescents with HFA may benefit more from programs focusing on emotional awareness, social cognition, and frustration tolerance, while those with CD may require more structured behavioral approaches aimed at impulse control and anger management.

## 5. Conclusions

The results of this study highlight that adolescents with high-functioning autism (HFA) exhibit significantly elevated anger profiles compared to typically developing peers, and in some dimensions, adolescents with HFA exhibited elevated scores that approached, but did not reach, those seen in conduct disorder. While the levels of anger in HFA do not reach the intensity of those with CD, the findings indicate notable emotional vulnerability among autistic adolescents, especially when confronted with interpersonal stress or criticism. This profile underscores the need for targeted emotion regulation interventions in this population, not only to manage aggression but also to enhance social adaptation and overall functioning.

Importantly, the difficulties with anger regulation identified in the HFA group are not always expressed through overt physical aggression—especially when compared to adolescents with conduct disorder, who showed significantly higher levels of physical anger expression, but rather through verbal expression and internalized emotional tension. This can lead to social or academic dysfunctions. These results reinforce the understanding of emotion regulation as a construct that necessitates individualized therapeutic approaches, irrespective of the presence or absence of overt aggressive behaviors.

### 5.1. Practical Implications

These findings carry important implications for mental health professionals and all those working with adolescents on the autism spectrum. Psychologists, psychiatrists, and therapists should be particularly attentive to the signs of emotional dysregulation—especially verbal anger and frustration reactivity—which may not always be overt or disruptive, but can still indicate underlying vulnerability to aggressive behavior. Early screening for emotion regulation difficulties and anger-related symptoms is essential for identifying individuals at risk and implementing timely, targeted interventions. Preventive strategies, including cognitive-behavioral therapy (CBT) programs and emotion regulation training, can help reduce the escalation of aggressive tendencies and support overall psychological adjustment.

In parallel, educators also play a crucial role in identifying and supporting students who exhibit emotional dysregulation in classroom settings. Integrating structured emotion regulation modules, social-emotional learning, and anger management techniques into school programs can empower autistic students to recognize and manage early signs of distress. Close collaboration between schools, families, and clinical specialists enhances the consistency of interventions across environments. By proactively addressing frustration tolerance and communication around emotional needs, professionals from both clinical and educational domains can contribute to safer, more inclusive, and developmentally responsive support systems for adolescents with autism spectrum disorder.

### 5.2. Study Limitations

This study presents several limitations. First, the relatively small sample size (n = 40 per group) reduces statistical power and limits the generalizability of the findings to broader clinical populations. Given the exploratory nature and sample size limitations, the present investigation should be considered a pilot study. Its findings provide a preliminary framework for future, larger-scale research into emotion regulation in adolescents with high-functioning autism. Second, the use of convenience sampling may introduce selection bias. Third, although we included an exploratory analysis of gender differences in anger expression and regulation, only one statistically significant difference emerged (on the Ac_o scale), and other patterns remained non-significant. Given the small subgroup sizes, these results should be interpreted with caution. Nonetheless, the limited representation and statistical power for detecting sex-based effects may have obscured meaningful gender-specific differences, especially in light of prior literature suggesting distinct emotional profiles in males and females with ASD or CD. Fourth, although adolescents with ADHD were excluded to minimize potential confounding and increase internal validity, this decision also restricts the applicability of the findings to the broader clinical population, in which ADHD is a frequent comorbidity. Additionally, reliance on self-report questionnaires may have introduced bias, particularly given known challenges with emotional insight and reporting accuracy in autistic individuals. Finally, the cross-sectional design of the study limits our ability to assess changes over time or the effects of interventions on anger regulation.

### 5.3. Future Research Directions

Future studies should aim to recruit larger and more diverse samples, including participants from non-clinical settings and those with common comorbidities such as ADHD. Longitudinal research is also necessary to explore developmental trajectories of anger regulation in HFA and to determine how these emotional challenges change with age or in response to targeted interventions. Moreover, the integration of neurobiological measures (e.g., functional connectivity studies, physiological markers of arousal) could provide a deeper understanding of the mechanisms underlying emotion dysregulation in autism. Investigating the efficacy of emotion regulation training, such as cognitive-behavioral or mindfulness-based therapies tailored for autistic youth, represents another promising avenue to improve psychosocial outcomes in this population.

## Figures and Tables

**Table 1:** Pairwise group comparisons on STAXI state anger scale using Mann–Whitney U test.

Scale	Comparison	Mean Rank HFA	Mean Rank CD	Mean Rank Control	U-Statistic	*p*-Value
S_ang	HFA vs. CD	33.63	47.38		525.00	0.008
S_ang	HFA vs. control	51.78		29.23	349.00	<0.001
S_ang	CD vs. control		60.15	20.85	14.00	<0.001
S_ang_f	HFA vs. CD	33.88	47.13		535.00	0.010
S_ang_f	HFA vs. control	52.61		28.39	315.50	<0.001
S_ang_f	CD vs. control		59.70	21.30	32.00	<0.001
S_ang_v	HFA vs. CD	35.60	45.40		604.00	0.058
S_ang_v	HFA vs. control	50.56		30.44	397.50	<0.001
S_ang_v	CD vs. control		59.04	21.96	58.50	<0.001
S_ang_p	HFA vs. CD	31.59	49.41		443.50	0.001
S_ang_p	HFA vs. control	46.66		34.34	535.50	0.017
S_ang_p	CD vs. control		58.69	22.31	72.50	<0.001

**Table 2:** Pairwise group comparisons on STAXI trait anger scale using Mann–Whitney U test.

Scale	Comparison	Mean Rank HFA	Mean Rank CD	Mean Rank Control	U-Statistic	*p*-Value
T_ang	HFA vs. CD	36.06	44.94		622.50	0.086
T_ang	HFA vs. control	52.68		28.33	313.00	<0.001
T_ang	CD vs. control		59.59	21.41	36.50	<0.001
T_ang_t	HFA vs. CD	39.06	41.94		742.50	0.575
T_ang_t	HFA vs. control	50.31		30.69	407.50	<0.001
T_ang_t	CD vs. control		56.80	24.20	148.00	<0.001
T_ang_r	HFA vs. CD	33.03	47.98		501.00	0.004
T_ang_r	HFA vs. control	52.28		28.73	329.00	<0.001
T_ang_r	CD vs. control		58.54	22.46	78.50	<0.001

**Table 3:** Pairwise group comparisons on STAXI anger regulation and expression scales using Mann–Whitney U test.

Scale	Comparison	Mean Rank HFA	Mean Rank CD	Mean Rank Control	U-Statistic	*p*-Value
Ax_o	HFA vs. CD	39.19	41.81		747.50	0.610
Ax_o	HFA vs. control	48.21		32.79	491.50	0.003
Ax_o	CD vs. control		53.08	27.93	297.00	<0.001
Ax_i	HFA vs. CD	38.03	42.98		701.00	0.338
Ax_i	HFA vs. control	44.23		36.78	651.00	0.148
Ax_i	CD vs. control	50.34		30.66	406.50	<0.001
Ac_o	HFA vs. CD	34.70	46.30		568.00	0.025
Ac_o	HFA vs. control	37.75		43.25	690.00	0.286
Ac_o	CD vs. control		47.08	33.93	537.00	0.010
Ac_i	HFA vs. CD	34.44	46.56		557.50	0.019
Ac_i	HFA vs. control	40.59		40.41	796.50	0.973
Ac_i	CD vs. control		50.39	30.61	404.50	<0.001
Ax_index	HFA vs. CD	46.05	34.95		578.00	0.032
Ax_index	HFA vs. control	49.04		31.96	458.50	0.001
Ax_index	CD vs. control		41.18	39.83	773.00	0.794

**Table 4:** STAXI-2 gender differences—significant results.

Group	Scale	*p*	Mean Rank Males	Mean Rank Females
HFA	Ac_o	0.047	23.17	15.54
CD	Ac_i	0.023	23.9	15.41
Control	S_ang_f	0.043	23.63	16.26
Control	T_ang	0.022	16.91	25.35
Control	T_ang_r	0.041	17.35	24.76
Control	Ac_i	0.009	16.43	26
Control	Ax_index	0.021	24.13	15.59

## Data Availability

The raw data supporting the conclusions of this article will be made available by the authors on request.
